# Epstein–Barr virus–host cell interactions: an epigenetic dialog?

**DOI:** 10.3389/fgene.2014.00367

**Published:** 2014-10-21

**Authors:** Hans H. Niller, Kalman Szenthe, Janos Minarovits

**Affiliations:** ^1^Institute of Medical Microbiology and Hygiene, University of Regensburg, Regensburg, Germany; ^2^RT-Europe Nonprofit Research Ltd, Mosonmagyaróvár, Hungary; ^3^Department of Oral Biology and Experimental Dental Research, Faculty of Dentistry, University of Szeged, Szeged, Hungary

**Keywords:** chromatin, CpG island, epigenome, viral latency, lytic cycle, pioneer transcription factor, tumor suppressor gene, tumor virus

## Abstract

Here, we wish to highlight the genetic exchange and epigenetic interactions between Epstein–Barr virus (EBV) and its host. EBV is associated with diverse lymphoid and epithelial malignancies. Their molecular pathogenesis is accompanied by epigenetic alterations which are distinct for each of them. While lymphoblastoid cell lines derived from B cells transformed by EBV *in vitro* are characterized by a massive demethylation and euchromatinization of the viral and cellular genomes, the primarily malignant lymphoid tumor Burkitt’s lymphoma and the epithelial tumors nasopharyngeal carcinoma and EBV-associated gastric carcinoma are characterized by hypermethylation of a multitude of cellular tumor suppressor gene loci and of the viral genomes. In some cases, the viral latency and oncoproteins including the latent membrane proteins LMP1 and LMP2A and several nuclear antigens affect the level of cellular DNA methyltransferases or interact with the histone modifying machinery. Specific molecular mechanisms of the epigenetic dialog between virus and host cell remain to be elucidated.

## EPSTEIN–BARR VIRUS—THE FIRST HUMAN TUMOR VIRUS

Epstein–Barr virus (EBV), the proto-typical gamma-herpesvirus infecting humans, has been discovered 50 years ago in cultured Burkitt’s lymphoma (BL) cells (Epstein et al., 1964). EBV physiologically homes to memory B cells, a property which is reflected in the genus name *Lymphocryptovirus* for gamma-herpesviruses which “hide in lymphoid cells.” EBV is the causative agent of infectious mononucleosis, and was the first known human tumor virus. In addition to BL, an endemic childhood tumor mainly of equatorial Africa, EBV plays a role in the origin or progression of other primarily malignant B cell tumors, such as Hodgkin lymphoma, and diverse AIDS-associated lymphomas including lymphomas of the central nervous system. A profound failure of T cell surveillance may allow early-onset post-transplant lymphoproliferative disorder (PTLD) which may be overcome upon a timely onset of T cell control, but may turn malignant if growing uncontrolled for too long a time span.

In the first decade of EBV research, EBV was considered a purely lymphotropic virus. Finding viral DNA in cellular DNA from biopsies of anaplastic carcinomas of the nasopharynx (NPC, nasopharyngeal carcinoma) by DNA hybridization did not change that general view, because NPC as a lymphoepithelial tumor contains a great many of infiltrating lymphocytes (zur Hausen et al., 1970). Localizing the virus specifically to the malignant epithelial cells first established EBV infection of non-lymphatic cells and paved the way for the novel concept of EBV as an epithelial tumor virus (Wolf et al., 1973). NPC is an endemic tumor with a strong preference for South East Asia, especially Guangdong and Hong Kong, with an incidence rate of 20–30 cases per 100,000 persons per year, and virtually 100% of non-keratinizing and undifferentiated NPCs are EBV-associated. Almost two decades later on, the association of EBV with gastric carcinomas (EBVaGC) was established, too (Burke et al., 1990; Shibata et al., 1991). Contrary to the endemic tumor NPC, about 10% of the worldwide sporadic tumor gastric carcinoma (GC) are associated with EBV infection. Among gastric remnant carcinomas approximately 30% and among lymphoepithelioma-like GCs, approximately 80% are EBV-associated. Altogether, EBVaGC with an estimated more than 80,000 cases per year is probably the most frequent EBV-associated malignancy worldwide (reviewed in Niller et al., 2014a,b). Beyond the B cell lineage, EBV infection is currently associated with T cell lymphomas, epithelial tumors, and rarely with leiomyosarcoma, a neoplasm of mesodermal origin (McClain et al., 1995). Furthermore, the risk of autoimmune disease, including multiple sclerosis, is significantly increased after primary EBV-infection, and even more so after symptomatic primary infection, i.e., mononucleosis (Niller et al., 2008).

## PATHOGENESIS OF EBV-ASSOCIATED TUMORS

The spectacular ability of EBV to transform and immortalize B cells dominated the first four decades of EBV research and tumor virology. General view was that the EBV-transformed cell was the origin of the endemic BL cell, too, although a fundamental difference of the epidemiology and pathogenesis between lymphoblastoid cell line (LCL)-like tumors on one side (early onset PTLD), and of primarily malignant EBV-associated lymphomas on the other side (endemic BL) became evident. Early onset PTLD originate under conditions of severe immune suppression and depend on viral transforming functions, including EBV nuclear antigens (EBNAs) and latent membrane proteins (LMPs) that are expressed both in PTLDs *in vivo* and in LCLs immortalized *in vitro*. On the other hand, BL and Hodgkin lymphoma originate under conditions of hyperstimulation of the lymphoid germinal center reaction, and they do not depend on EBNA2 which the vast majority of them do not express (Klein, 1987; Lenoir and Bornkamm, 1987; Table 1). In this context, it is important to distinguish between morphological and oncogenic transformation (Niller et al., 2011). Our discovery of a binding site for the oncoprotein c-Myc in the central locus control region of the EBV genome suggested that the molecular pathogenesis of endemic BL does not depend on a previous EBNA2-transformed state of the B cell, but mostly on a dysbalance of pro- and anti-apoptotic functions in consequence of Myc-translocation, a molecular accident in a virus-infected B cell undergoing the germinal center reaction (Niller et al., 2003). The need to counter-balance the pro-apoptotic force of translocated *c-Myc* through anti-apoptotic functions, either encoded by the viral genome or induced by virus infection, in order for a BL to emerge has recently been re-emphasized (Mbulaiteye, 2013; Westhoff Smith and Sugden, 2013; Rickinson, 2014). Our differential pathogenesis model for EBV-associated lymphomas was controversial at first (Rossi and Bonetti, 2004; Thorley-Lawson, 2004; reviewed in Niller et al., 2012), but has now gained strong support by recent large-scale epigenomic analyses of LCLs and tumor cells (see below). Thus, finding the binding site for the oncoprotein c-Myc in the locus control region of EBV caused a conceptual shift away from the morphologically transformed cell and has turned out as a heuristic discovery (Niller et al., 2003).

**Table 1:** **Host cell-dependent expression of latent Epstein–Barr virus proteins.**

Protein	Cell type and latency type			
	BL	NPC	CLL	LCL
	Type I	Type IIa	Type IIb	Type III
EBNA1	+	+	+	+
EBNA2	–	–	+	+
LMP1	–	+/–	–	+
LMP2A	–	+	+	+
BARF1	–	+	–	–

Key viral latency proteins or so-called oncoproteins are differentially expressed in EBV-associated malignancies and transformed cell lines. Depending on the specific viral gene expression patterns, specific viral latency classes are defined. BL, Burkitt’s lymphoma; NPC, nasopharyngeal carcinoma; CLL, chronic lymphocytic leukemia; LCL, lymphoblastoid cell line; EBNA, Epstein–Barr virus nuclear antigen; LMP, latent membrane protein; BARF1, BamHI A rightward frame 1.

## GENETIC EXCHANGE BETWEEN HERPESVIRUSES AND HOST CELLS

Homology between herpesviral and human genes is now a common theme which was first highlighted by the finding of a gene for a functional thymidylate synthase (TS) in the genome of herpesvirus saimiri with an extremely high homology of 70% identical amino acids with the human *TS* gene. Various parameters suggested that the *TS* gene had been acquired in virus evolution by an ancestral herpesvirus from the cellular genome (Bodemer et al., 1986; Honess et al., 1986). The exchange of human and viral genes, and in the case of human herpesvirus 6 (HHV-6) the invasion of an entire herpesviral genome into the human germ line (Daibata et al., 1999) in about 0.8% of humans, must have happened on numerous occasions in evolutionary time. In the case of EBV, the intimate evolutionary relationship of virus and host cell is emphasized by the presence of several viral genes with sequence homology to cellular genes, i.e., *BHRF1* and *BALF1*, two anti-apoptotic *BCL2* homologs, *BILF1*, coding for a constitutively active G protein-coupled receptor (GPCR) homolog, and *BCRF1*, an *IL-10* (*interleukin 10*) gene homolog. The BCRF1 protein appears to be a functional homolog of IL-10, an immune suppressive cytokine secreted by Th2 cells with a sequence identity of about 70% (Hsu et al., 1990). The sequence homologies of the other three viral peptides are of lower degree than that. BHRF1 carries a 25% identity of a 150 amino acid C-terminal portion with the anti-apoptotic cellular protein BCL2 (Cleary et al., 1986). BALF1 shows homology at a similar degree in functionally important domains to its cellular counterparts BCL2 and BCLX (Marshall et al., 1999). BILF1 carries a homology of around 20% with the human chemokine receptor CXCR3A (Davis-Poynter and Farrell, 1996; Beisser et al., 2005; Paulsen et al., 2005). EBV-associated carcinoma cells express BARF1, identified initially as a lytic cycle protein that binds to hCSF-1 (human colony stimulating factor 1) as a viral decoy receptor, although its crystal structure is most closely related to CD80, a co-stimulatory molecule of antigen presenting cells (Seto et al., 2005; Tarbouriech et al., 2006; Elegheert et al., 2012).

Herpesviral DNA-binding proteins ICP8 of HSV1 and BALF2 of EBV, and additional homologous proteins of human herpesviruses which are required for viral replication belong to a class of “DDE/RNase H-like fold-family” nucleases, together with the recombination activating gene (RAG) 1 protein, essential for V(D)J recombination, and the Argonaute protein of the RNA-induced silencing complex (RISC). For ICP8 of HSV, divalent cation binding of the DDE-site was actually shown to be functional and required for viral replication (Bryant et al., 2012). Furthermore, inhibitors of HIV integrase, another RNase H-fold protein, inhibited the replication of viruses from all herpesvirus genera, too (Yan et al., 2014). Based on a co-regulatory transcriptional network for both *RAG-1* and *RAG-2*, and the genes for herpesviral DNA-binding proteins, and based on signature sequence homologies between *V(D)J* recombination sites and the viral terminal repeats, an evolutionary relationship between the RAG recombinase and herpesviral DNA-binding proteins was proposed. The *RAG* locus may have originally been introduced to host cells by a primordial herpesvirus (Dreyfus, 2009). We found a striking co-linearity of structural and functional elements between the cellular immunoglobulin gene loci and the left part of the EBV genome. Therefore, although speculative, we agree with the view that, in the case of the *RAG* genes, the appearance of the adaptive immune system may have been dependent on a primordial herpesvirus genome and, in the case of the B cell, may have developed further as consequence of an evolutionary ping-pong game between EBV and the host cell (Niller et al., 2004).

## EPIGENETIC INTERACTIONS BETWEEN EBV AND ITS HOST CELLS

Epstein–Barr virus infects both B lymphocytes and epithelial cells *in vivo*. It enters B cells after binding to CD21, a cell surface molecule absent from epithelial cells. However, EBV-infected B cells are capable to transfer the virus to epithelial cells lacking the EBV receptor. Both B cells and epithelial cells can support productive (lytic) EBV replication when all of the proteins and non-translated RNAs encoded by the viral genome are expressed in a sequential order. EBV also causes latent infections, typically in resting memory B cells and in various neoplastic cells that usually carry circular, double stranded viral genomes. During latency, only a restricted set of EBV promoters is active. The activity of latent EBV promoters depends on the phenotype of host cells, and it is controlled by the epigenetic regulatory machinery. Based on the epigenetic marks deposited on the viral chromatin by the cellular epigenetic machinery one can distinguish between viral epigenotypes that are associated with unique patterns of viral gene expression (reviewed in Minarovits, 2006). In parallel, certain latent EBV proteins characteristic for the major cell types carrying latent EBV genomes act as epigenetic regulators themselves: they alter the cellular epigenotype and gene expression pattern and may contribute to the development of malignant tumors. Thus, the situation is similar to an “epigenetic dialog,” indeed. Typical examples of EBV latency types are summarized in Table 1, based on the nomenclature suggested by Klein et al. (2013; see also Laytragoon-Lewin et al., 1995; Niller et al., 2012).

## EPIGENETIC CONTROL OF EBV LATENCY PROMOTERS

The epigenetic regulatory mechanisms of host cells not only control the preservation of cell type-specific gene expression patterns from cell generation to cell generation, but ensure the maintenance of host cell-dependent usage of latent EBV promoters as well. Epigenetic regulation is based on writing, reading, and erasing epigenetic marks on chromatin as well as on protein–DNA interactions that are stable even in mitotic, highly condensed chromatin (reviewed in Gopalakrishnan et al., 2008; Zaret et al., 2008; Sharma et al., 2010; Blomen and Boonstra, 2011). Euchromatic marks favor transcription whereas heterochromatic marks are associated with a more condensed chromatin structure that usually represses promoter activity.

DNA methylation is involved in silencing of most latent EBV promoters. It is well documented that the alternative promoters Wp and Cp, where transcripts coding for six EBNAs are initiated can be switched off by CpG methylation. In addition, LMP1p and LMP2Ap, the promoters for LMP1 and LMP2A transcripts are silenced by the activity of cellular DNA methyltransferases, too. DNA methylation does not play a role, however, in switching off Qp, an alternative promoter for EBNA1 transcripts (reviewed in Li and Minarovits, 2003; Niller et al., 2012). One may speculate, that EBNA2, the major transactivator protein encoded by the EBV genome that interacts with both histone acetyltransferases and histone deacetylases (reviewed in Niller et al., 2009) may activate key cellular genes mediating genome-wide demethylation in LCLs. Acetylation of histone H3 and histone H4 molecules and di- or trimethylation of lysine 4 of histone H3 (H3K4me2 or H3K4me3) are euchromatic marks frequently associated with active Cp, Qp, LMP1p, and LMP2Ap (reviewed in Niller et al., 2012; Arvey et al., 2013). In principle, complexes formed by Polycomb and Trithorax group proteins that modify histone tails could also play a role in the regulation of latent EBV promoters but there are no data supporting such a mechanism. In contrast, Polycomb group protein EZH2 was observed to leave a heterochromatic histone mark (H3K27me3) at early lytic promoters of EBV that are silent during latency, and chromatin immunoprecipitation proved the association of the EZH2 methyltransferase with this class of viral promoters in the BL cell line Raji (Woellmer et al., 2012). The immediate-early promoter Zp, where transcripts for the lytic cycle initiating BZLF1 protein are initiated was also found to be repressed by H3K27me3 but also by H4K20me3 in Raji cells (Murata et al., 2012).

Pioneer transcription factors and variant histone molecules that bind to repressive chromatin areas and mark the genes to be activated have not been implicated in the control of EBV latency. Chromatin loops formed by binding of insulator proteins to EBV episomes may play a role, however, in the epigenetic regulation of the latency promoters Cp and Qp (Tempera et al., 2011).

## EBV-ENCODED ONCOPROTEINS AS EPIGENETIC REGULATORS

The methylation patterns of NPC cells and EBVaGC cells differ from their normal counterparts: these neoplastic cells regularly carry hypermethylated genomic regions with silenced cellular promoters (Iizasa et al., 2012; Lo et al., 2012). Focal hypermethylations frequently inactivate tumor suppressor genes and may contribute to carcinogenesis and tumor progression. Because the EBV-encoded transmembrane proteins LMP1 and LMP2A are capable of upregulating the cellular DNA methyltransferases DNMT1, DNMT3A, and DNMT3B, it was suggested that hypermethylation of CpG rich sequences, the so called CpG islands, is mediated by LMP1 or LMP2A in EBV-associated carcinomas (reviewed in Niller et al., 2012). LMP1 and LMP2A are expressed in EBV positive Hodgkin lymphomas as well. Thus, they may contribute to gene silencing in these neoplasms, too.

It is worthy to note that, similarly to the EBV-associated carcinomas, LCLs established by *in vitro* EBV-infection of B cells also express LMP1 and LMP2A that could potentially upregulate DNA methyltransferases. It was observed, however, that the typical epigenetic change in LCLs is a widespread demethylation of the B cell methylome affecting one third of all cellular genes and 2.18 GB of the genome (Hansen et al., 2014). The mechanism of demethylation remains to be established. Furthermore, the EBV episomes carried by LCLs are also hypomethylated, in contrast to the overall hypermethylation of EBV genomes in latency type I BL lines, BL biopsies, and EBV-associated carcinomas (Minarovits et al., 1991; Fernandez et al., 2009). The viral oncoproteins EBNA3A and EBNA3C expressed during *in vitro* immortalization of B cells silence distinct cellular tumor suppressor genes by depositing a heterochromatic histone mark via the Polycomb repressor complex PCR2 (reviewed in Allday, 2013). The dominant change in EBV immortalized B cells seems to be, however, a genome-wide decrease and redistribution of heterochromatic marks (Hernando et al., 2014). The EBV latency products eliciting the reprograming of the host cell epigenome in LCLs remain to be elucidated. Contrary to LCLs, primarily malignant lymphomas, i.e., BL and other lymphomas, are characterized not by a massive and wide-spread hypomethylation, but by a local hypermethylation of selected genomic loci (Martin-Subero et al., 2009a,b; Kreck et al., 2013; reviewed in Niller et al., 2014b).

In contrast to BL tumors, in which hypermethylated loci are strongly enriched for polycomb repressive complex (PRC) target genes of embryonic stem cells, hypermethylated genes in EBVaGC are not enriched for PRC targets (Martin-Subero et al., 2009a; Matsusaka et al., 2011). Contrary to *Helicobacter pylori*-associated GC, EBVaGC does not emerge from an “epigenetic field” in the gastric mucosa (Matsusaka et al., 2011; Niller et al., 2014a). Thus, EBV-associated epigenetic changes may quite quickly set the stage for malignancy (Au et al., 2005; Niller et al., 2014a). Notably, even transient EBV infection of epithelial cells leaves permanent epigenetic scars indicating past infection (Queen et al., 2013; Birdwell et al., 2014).

Although there are no data as to the interaction of host cell-encoded pioneer transcription factors and the EBV genome, certain viral proteins that bind to both viral and cellular DNA and remain associated with mitotic chromosomes may act as pioneer transcription factors. EBNA1, a nuclear protein expressed in all EBV latency types is a putative pioneer factor functionally resembling transcription factors of the FoxA family that act as epigenetic regulators controlling important developmental processes (Niller et al., 2012). EBNA1 was shown to bind to different sets of cellular promoters in cell lines of epithelial and lymphoid origin, and upregulation as well as downregulation of distinct gene batteries was observed (Canaan et al., 2009). In the BL cell line Raji, EBNA1 was shown to interact with a large number of cellular genes as well as LINE elements, and high affinity EBNA-binding sites were observed in a repetitive sequence in chromosome 11 (Lu et al., 2010).

BZLF1, an immediate-early EBV protein initiating productive viral replication that preferentially binds methylated DNA sequences was also suggested to act as a pioneer transcription factor (Woellmer et al., 2012). BZLF1 is expressed not only during the lytic cycle but transiently also during the establishment of latent infection of B cells *in vitro* (Kalla and Hammerschmidt, 2012). Because BZLF1 can bind to cellular promoters (Lan et al., 2013) and elicit epigenetic alterations (Woellmer et al., 2012), an “epigenetic dialog” between the latent EBV genomes and the host cell genome may occur, indeed: transient BZLF1 expression may change the cellular epigenotype followed by silencing of the BZLF1 promoter through the cellular epigenetic machinery. In parallel, EBV-encoded epigenetic regulators may leave their marks on the cellular epigenotype in a next phase of EBV-mediated B cell immortalization.

In conclusion, on an evolutionary time scale a genetic exchange between herpesviruses and their hosts is evident. Beginning with the early steps of viral infection, epigenetic interactions between virus and host cell are taking place. The multi-tiered epigenetic dialog between EBV and its host needs to be elucidated in greater molecular detail in order to understand the diverse outcomes of infection.

### Conflict of Interest Statement

The authors declare that the research was conducted in the absence of any commercial or financial relationships that could be construed as a potential conflict of interest.
